# Improved Oxidative Stability by Embedded Cerium into Graphene Oxide Nanosheets for Proton Exchange Membrane Fuel Cell Application

**DOI:** 10.3390/membranes11040238

**Published:** 2021-03-28

**Authors:** Prem P. Sharma, Vo Dinh Cong Tinh, Dukjoon Kim

**Affiliations:** School of Chemical Engineering, Sungkyunkwan University, Suwon 440-746, Gyeonggi, Korea; premsharma15@gmail.com (P.P.S.); vodinhcongtinh@gmail.com (V.D.C.T.)

**Keywords:** hybrid material, GO, proton exchange membrane fuel cell (PEMFC), radical scavenger

## Abstract

Investigation of the collaborative effect of cerium particles embedded in graphene oxide to enhance the chemical stability of a proton exchange membrane fuel cell (PEMFC) has been carried out. Synthesis of composite membranes (Nafion-GO/Ce-x) with Nafion solution as a polymer is synthesized by a solution casting method where (x = concentration of composite). The developed hybrid material was characterized by FT-IR and X-ray diffraction (XRD) for its phase identification while the chemical structure was characterized by XPS analysis. The enhancement in the chemical stability of the incorporated hybrid material is characterized by Fenton’s test showing a radical scavenging effect. It was found that the residual weight for Nafion ^212^ was 92.50% after 24 h and it was 94.32% for Nafion-GO/Ce-2 and 96.49% for Nafion-GO/Ce-4, proving the suitability of composite membranes for fuel cell applications.

## 1. Introduction

Due to their advantages of high energy density and low emission of pollutants (SOx, NOx), proton exchange membrane fuel cells have received significant attention due to their potential as a clean and efficient source for energy production [1,2,3,4], although their commercial usage is still restricted because of some technical obstacles. The most common issue is the poor durability of the core material in the form of an electrolyte membrane in the fuel cell system [5]. For a proton exchange membrane to be used in fuel cell, it should possess high chemical stability and mechanical stability as well as excellent dimensional stability with high proton conductivity [6,7]. Nafion, a perfluorosulfonated acid polymer, is widely used in proton exchange membranes for fuel cell technology but its long-term stability remains poor [8,9]. In order to address the above problems, new materials are being generating by several research institutions to improve the chemical and mechanical stability of the membranes for fuel cell technology [10].

Studies have been conducted to investigate the degradation mechanism behind the repeated attack of HO**·** formed due to the reaction between H_2_O_2_ and precious transition metals [11,12]. The vulnerable sites in the polymer electrolyte membrane are –COOH, –C–O–C– (ether bond) and –C–S– (sulfide bond), which is mainly counterattacked by radicals to release –CO_2_ and HF as side products [13,14]. Several approaches have been introduced to improve the chemical stability of the polymer electrolyte membrane, including the involvement of radical quenchers from organic/inorganic molecules, metal ions and organic/inorganic nanoparticles [15,16]. The properties of Nafion that contribute to its chemical stability were investigated by the introduction of multiwalled carbon nanotubes (MWCNT), zirconia phosphate and titania, which mainly functioned as organic and inorganic fillers [17]. They resulted in a significant increase in the stability but polymer electrolyte membranes still suffer from low proton conductivity. On the other hand, use of metal ions such as Mn^+2^ is also able to achieve good chemical stability, but studies demonstrate that it is able to scavenge only 46% of the radicals (HO**·**) generated during the fuel cell operation [18].

Cerium ion is considered the best metal ion as a radical scavenger as it bears a unique ability to convert Ce^+3^ To Ce^+4^ when exposed to acidic medium and can generate Ce^+3^ when it is reacted with H_2_O_2_ [19]. It could increase the antioxidation stability of the polymer electrolyte membrane, which may increase the durability for long-term operation, but the excess amounts of polymer could result in a decrease in proton conductivity due to the neutralization of sulfonic acid groups with the positive charge of cerium [20]. On the other hand, water is a solvent for Ce ions; thus, it could leach out from the membrane. Therefore, this issue can be overcome by establishing an indirect ionic interaction between Ce ion with that of the acid groups in polymers to achieve maximum chemical stability for long-term operation.

The current study was carried out for the synthesis of a stable composite proton exchange membrane having radical scavenging ability in a harsh oxidative environment. GO was chosen to accommodate cerium, so as to avoid the direct interaction of the functional groups present on the polymer backbone to stabilize the proton conductivity to an extent. Further, systematic study of the physicochemical properties was conducted to investigate the effect of the hybrid material (GO/Ce) on the polymer backbone. Moreover, synthesized membranes were subjected to Fenton’s test to reveal the durability of the membranes for electrochemical applications.

## 2. Materials and Methods

### 2.1. Materials

Nafion solution (D521, 5 wt%, EW 1100) in a mixture of alcohol and water were purchased from Chemours (Wilmington, NC, USA). Cerium (III) nitrate hexahydrate and graphene oxide (GO) were purchased from Sigma Aldrich (Milwaukee, WI, USA). Dimethyl sulfoxide (DMSO) and platinum (nominally 40% on carbon black, HiSPEC 4000) were purchased from Alfa Aesar (Ward Hill, MA, USA), and 2-Propanol (IPA) was purchased from Samchun Pure Chemical Company (Seoul, Korea).

### 2.2. Synthesis of Hybrid GO/Ce Material

**Scheme 1:**
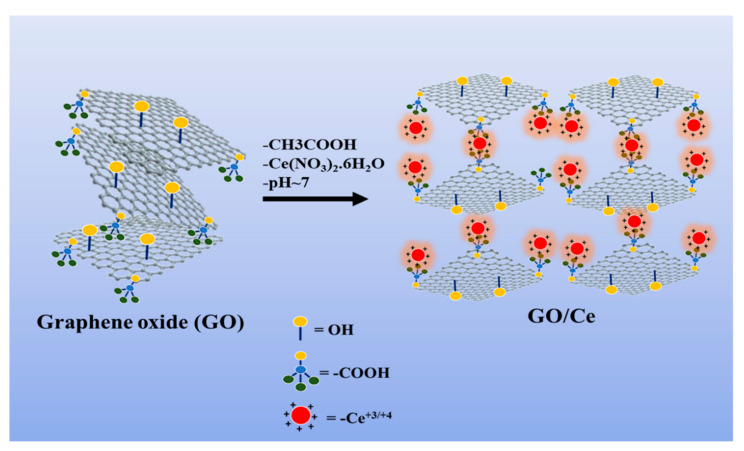
Illustration showing the synthesis of GO/Ce hybrid composite material from graphene oxide (GO).

For synthesis of GO/Ce-based hybrid material, 0.1 g of graphene oxide GO was dispersed in 100 mL pH-7 solution and subjected to low sonication for 30 min. Subsequently, 0.1 g of cerium nitrate hexahydrate was added to this solution, followed by adding 0.5 mL of CH_3_COOH solution and the mixture was subjected to 120 °C for 24 h in a Teflon-based autoclave. After completing the reaction, the solution was washed with ethanol several times and dried under vacuum oven at 60 °C for 12 h; see Scheme 1.

### 2.3. Synthesis of Composite Membrane

A predetermined amount of GO/Ce powder was first dispersed into IPA with the help of a sonicator for 30 min. Then, Nafion ^212^ solution (5 wt%) was added and the solution was subjected to sonication for 1 h followed by vigorous stirring at 150 rpm for 3 h. After obtaining complete homogeneous dispersion, the solution was cast on a clean glass plate and placed in the oven at 80 °C for 24 h and then at 120 °C for an hour under a vacuum oven. Synthesized membranes were peeled off and designated as Nafion-GO/Ce-x (where x 0.5, 1, 2 and 5 wt%). Afterward, these were dipped into 1 M H_2_SO_4_ solution for 24 h before further use.

### 2.4. Characterization

#### 2.4.1. Chemical Structure Analysis

Fourier-Transform Infrared Spectroscopy (FT-IR) spectra were measured using a Perkin-Elmer FT-IR Frontier instrument (Nicolet iS10, Thermo Fisher, Ward Hill, MA, USA). Phase identification for hybrid material was determined by X-ray diffraction (D8 Advance) with CuKα radiation (λ = 1.54 Å). The surface morphology and elemental analysis were evaluated by FE-SEM (EM, Phillip XL30 ESEM-FCG, North Billerica, MA, USA). Thermal stability of hybrid membranes was characterized by TGA analysis (TGA, Seiko Exstar 6000, Tokyo, Japan).

#### 2.4.2. Ion Exchange Capacity (IEC)

The IEC of the membranes was calculated by the acid-base titration method. The membrane samples were washed with DI water and completely dried to measure their weight (in gram) before being immersed in 1.0 M NaCl solution to complete the exchange of H^+^ ions into Na^+^ ions. The solution was then titrated with 0.1 M of NaOH solution using phenolphthalein as an indicator. The *IEC* (meq/g) values of membranes were calculated from Equation (1):

(1) $$IEC\;\left(\mathrm{meq}/g\right)=\frac{C_{NaOH}\times V_{NaOH\;}}{W_{dry}}$$

where *C_NaOH_* and *V_NaOH_* are the volume of titrated NaOH solution and *W_dry_* is the dry membrane weight.

#### 2.4.3. Proton Conductivity

The membranes were immersed in water and then cut into 3 cm (length) × 1 cm (width) sections having a thickness ~80 μm to measure proton conductivity. The sample was placed in a 4-probe cell (BEKKTECH, Loveland, CO, USA) and in-plane hydroxide ion conductivity was measured by alternating current (AC) impedance spectroscopy (Zahner IM6e, Kronach, Germany) with a frequency range from 1 Hz to 1 MHz at 5 mV under 100% relative humidity. The bulk resistance of the membrane was directly obtained from the impedance curve and the hydroxide ion conductivity of the membrane was determined from the resistance using Equation (2):

(2) $$\sigma \;=\;\frac{L}{R\;W\;T}$$

Here, σ is the hydroxide ion conductivity of the membrane in (S cm^−1^), L is the distance in the direction of the ion flow between the measurement probes in cm, R is the bulk resistance of the membrane in ohm, W is the width of the membrane in cm, and T is the thickness of the membrane in cm.

#### 2.4.4. Water Uptake and Swelling Ratio

Water uptake for all composite membranes was measured by dipping the membrane pieces of (2 × 2) cm into DI water for 24 h and then values were calculated by the following equation:

(3) $$WU\left(\%\right)=\frac{Wet_{w}-Dry_{w}}{Dry_{w}}$$

where *Wet_w_* is the weight of the wet membrane and *Dry_w_* is the weight of the dry membrane.

Moreover, swelling ratio was calculated from Equation (4):

(4) $$\mathrm{Swelling}\;\mathrm{ratio}\;(\%)=\;\frac{L_{s}{-\;L}_{d}}{L_{d}}$$

where *L_s_* is the length of the wet sample and *L_d_* is the length of the dry sample, respectively.

#### 2.4.5. Thermal and Chemical Stability

A thermogravimetric analyzer (TGA, Seiko Exstar 6000, Tokyo, Japan) was used to investigate the thermal stability of all synthesized membranes. The dry membrane sample was thermally scanned at a ramp rate of 10 °C min^−1^ from 30 to 800 °C under a nitrogen gas atmosphere.

#### 2.4.6. Oxidative Stability

Antioxidation possibility of pristine and hybrid samples was investigated by measuring the residual weight percentage of each after Fenton’s solution treatment. The completely dry membrane pieces were immersed in the Fenton’s solution (3 wt% H_2_O_2_, 4 ppm Fe^2+^) at 80 °C from 24 h. After this time, the sample pieces were washed several times with DI water and then dried at 80 °C. The residual weight percentage (*RW*) was calculated by the difference in the weight of the sample before (*m_b_*) and after treatment (*m_a_*) from Equation (5).

(5) $$RW\left(\%\right)=\frac{m_{a}}{m_{b}}\times 100$$

## 3. Results and Discussion

### 3.1. Chemical Structure Characterization

**Figure 1:**
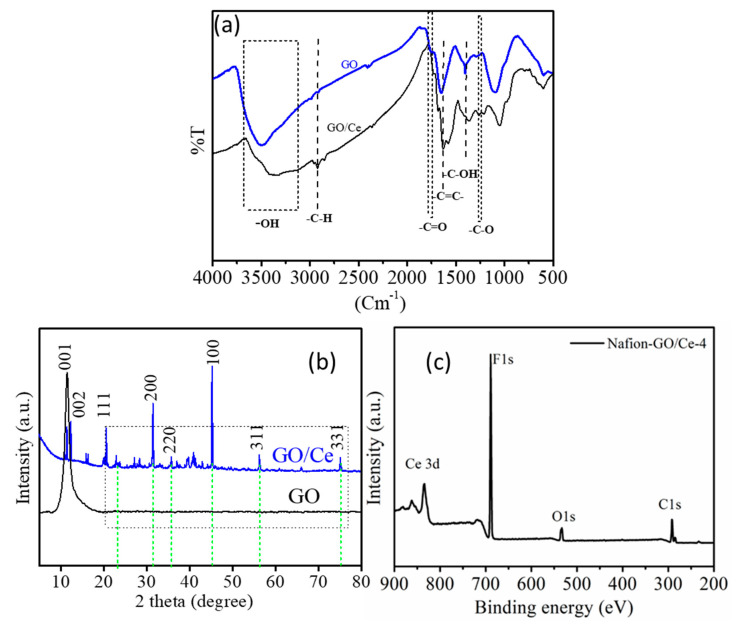
(**a**) FT−IR of GO and GO/Ce, (**b**) XRD spectra of GO and GO/Ce hybrid material, (**c**) XPS spectra of composite membrane.

The chemical structure characterization of both graphene oxide (GO) and GO/Ce was carried out by FT-IR, XRD and XPS analysis. In the FTIR spectrum, the characteristic peaks corresponding to the stretching vibration of –OH (3300–3000 cm^−1^), C–H (2930 cm^−1^), –C=C– (1643 cm^−1^) –C–O–C– (1253 cm^−1^) and –C=O (1753 cm^−1^) are related to the existence of oxygen functionalities present on the basal plane of the GO sheets [21,22]. The substantial reduction in bands at –C–O–C– (1253 cm^−1^) and –C=C (−1643 cm^−1^) in GO/Ce indicates the intercalation of cerium to the GO sheets. Moreover, the shifting of the band corresponding to 1653 cm^−1^ in the case of GO/Ce towards a higher wavenumber also suggests the weakening of aromatic ring stacking due to π–π stacking. There is a subsequent reduction in the region at 3300 cm^−1^ for GO/Ce because of neutralization of –OH associated with the –COOH group with Ce^+3/+4^; see Figure 1a. In the XRD study, an intense peak at 2θ = 11.2 associated with the (001) plane was attributed to the regular and continuously stacked sheets present in graphene oxide; see Figure 1b [23]. On the other hand, this peak is shifted to a higher value than that of the angle with broadening, showing that the complete reduction of the –COOH group and a slightly amorphous nature occurred due to the inner augmentation of cerium particles. Secondly, interplanar values at respective diffraction angles correspond to lattice planes (002), (111), (200), (220) (100) and (331), and (222) confirms the existence of cerium in GO, respectively [24].

Another confirmation of the presence of cerium (Ce 3d) in the composite membrane was shown by the XPS spectra, which yielded a value of binding energy of 880 eV and 516 eV for oxygen atoms; see Figure 1c [25].

**Figure 2:**
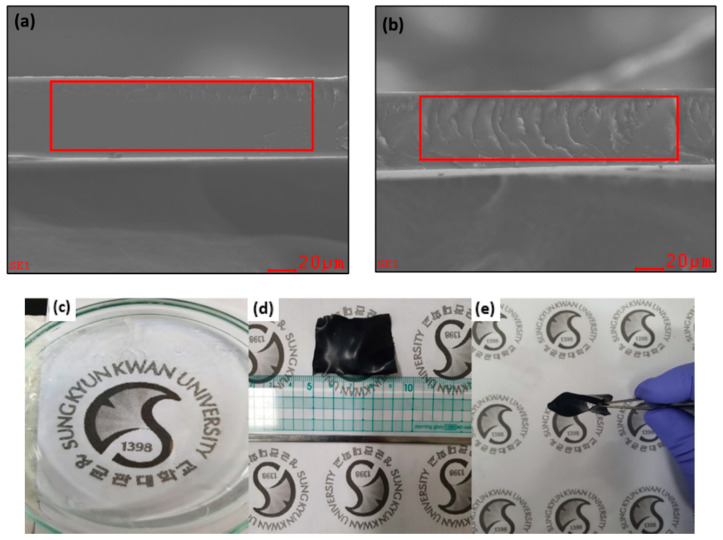
(**a**,**b**) FE-SEM images of pristine Nafion ^212^ and Nafion-GO/Ce (**c**) optical images of pristine and (**d**,**e**) composite membrane with the highest content.

The surface morphology was characterized by FE-SEM analysis, in which the cross-sectional study of the formed membrane showed its homogeneous and dense nature without any holes or cracks. Moreover, the wrinkled morphology observed in Figure 2b corresponds to the lamellar, layered structure of graphene oxide, whereas the surface morphology of the pristine membrane is smooth; see Figure 2a. The incorporation of the composite material to an extent does not destroy the inner morphology of the membrane. Optical photographs were also recorded for the synthesized composite membrane to show that there was no local agglomeration of the composite material.

### 3.2. Proton Conductivity, Water Uptake and Swelling Ratio

The efficient proton transportation of the polymer electrolyte membrane is a very useful parameter for fuel cell applications. The temperature-dependent proton conductivity was measured at a difference of 10 °C/min from 30 to 80 °C. A regular increment of proton conductivity was observed. The conductivity values of composite membranes are slightly lower than those of pristine membranes. The hydrogen bridge bonding formed between hydrophilic sites of graphene oxide with those of the functional group of Nafion would promote proton transfer by vehicular mechanisms.

**Figure 3:**
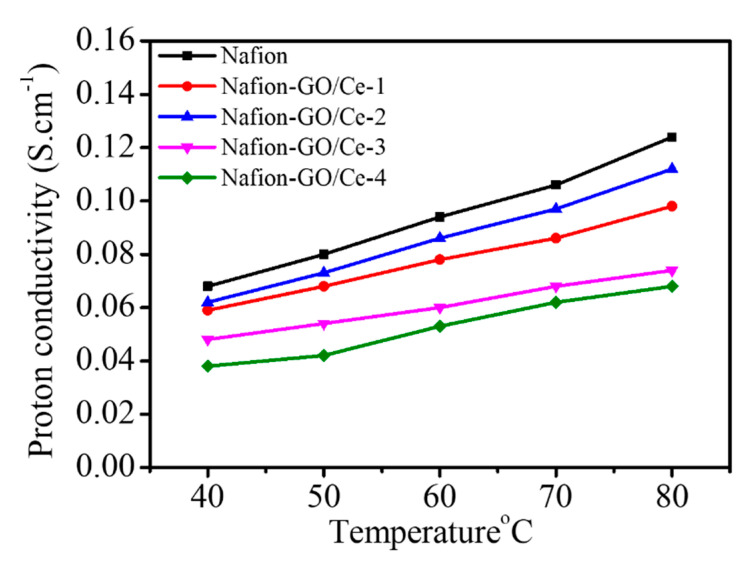
Proton conductivity vs. temperature of different composite membranes.

Water uptake and swelling ratio for a membrane are very crucial parameters for a polymer electrolyte membrane to be used for electrochemical applications; see Figure 3. The value for water uptake is higher for the composite membrane while it decreases for the pristine Nafion membrane because of the presence of fewer sites for bound water. As water is a polar solvent, it is attracted towards the polar group. However, as we incorporated cerium ions, the chance of a formed ionic interaction is preferably higher towards its ionic state. The water uptake value is lowest for Nafion-GO/Ce-4, which is due to possible agglomeration, which results in the formation of a blockage for bound water. The same trend is also shown by the IEC values for different composite membranes, and it is highest for pristine Nafion rather than Nafion-GO/Ce and Nafion/Ce [26,27].

**Figure 4:**
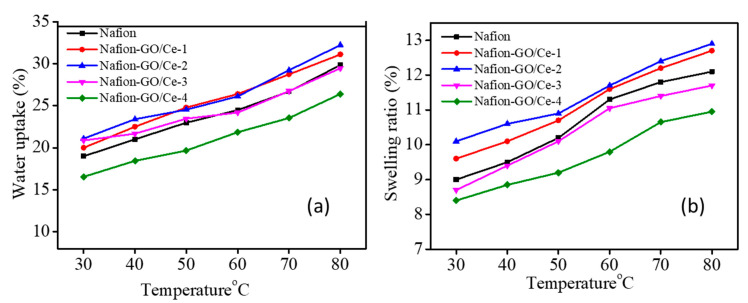
(**a**) Water uptake and (**b**) swelling ratio vs. temperature of different composite membranes.

Investigation of swelling ratio as a function of temperature also predicts the long-term usability of the polymer electrolyte membrane for fuel cell applications; see Figure 4b. In this case, the swelling found in Nafion-GO/Ce-2 is the highest as compared with the Nafion ^212^ and Nafion-GO/Ce-4. It was found to be 12% for Nafion at 80 °C and the lowest degree of swelling was found in Nafion-GO/Ce-4, which indicates the restricted movement of the polymer chain due to entanglement with the composite material. The intrinsic free volume inside the polymer restricts it expansion, thus reducing the swelling degree, which might possibly be reduced by the composite material; see Table 1.

**Figure 5:**
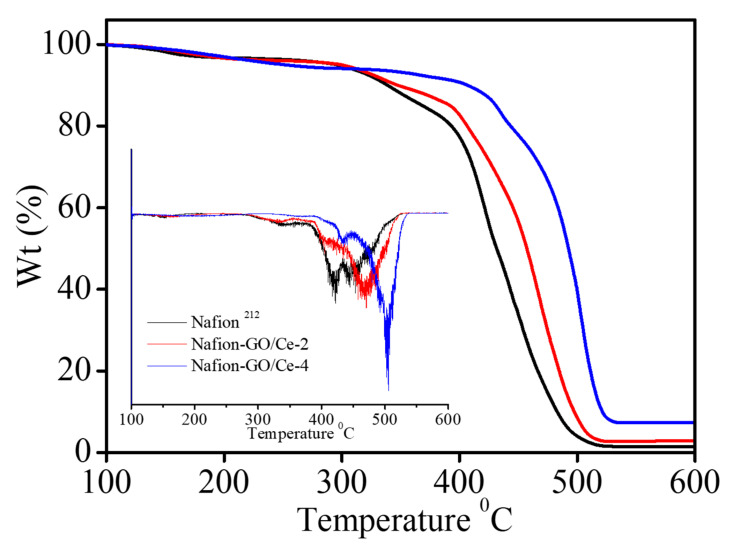
TGA and dTg (inset) graph of Nafion ^212^ and composite membranes.

The thermal study of all membranes was conducted by TGA analysis; see Figure 5. Three stages of weight loss occurred in the TGA graph at 100–150 °C, in which the first is associated with the evaporation of absorbed bound water content; see Figure 5. The second weight loss occurred in the range of 200–250 °C and is associated with the degradation of functional groups. In this region, the weight loss occurs to a larger extent in the case of composite membranes because of the presence of more functional groups on the surface of GO. On the other hand, the weight loss variation was greater in the region between 300 and 500 °C, because of the rapid extensive burning of the polymer, leaving the hybrid stable composite part, including GO. The final degradation occurred because of polymer backbone breakdown; the third step occurred in the region of 550 °C, and, from the TGA spectra, it can be seen that the degradation occurred at a higher range of temperatures in the case of Nafion-GO/Ce-4 due to the presence of thermally stable graphene oxide as a composite material. Additionally, dTg spectra also demonstrate stepwise weight loss (%) as a function of temperature. The higher temperature stability of the synthesized composite membrane proves its stability for use in high-temperature fuel cell systems.

The durability of the membrane was checked by Fenton’s test because the life of a membrane for fuel cell applications can be determined using this method. The severe attack of **·**OH and **·**OOH radicals is the main factor involved in the degradation of the polymer backbone [28,29,30]. Herein, it is hindered by inserting cerium ions into the membrane. The current approach has two advantages: (i) the ionic state of cerium ion is engaged by the -COOH bonds present on the surface of graphene oxide, due to which the proton conductivity is not decreased to a significant extent, and (ii) the leaching of cerium ion is supposed to be slow because of the physical interaction with the carboxylic acid present in the graphene oxide. The degradation was investigated by measuring the weight loss of the membranes before and after Fenton’s test. It was found that the residual weight for Nafion was 92.50% after 24 h and it was 94.32% for Nafion-GO-Ce-2 and 96.49% for Nafion-GO-Ce-4.

## 4. Conclusions

The successful intercalation of cerium into the aromatic sheets of graphene oxide is revealed by FTIR and XRD analysis, while the successful incorporation of a synthesized hybrid material (GO/Ce) into the polymer matrix (Nafion ^212^) is characterized by XPS analysis. The surface morphology of pristine and composite membranes with their elemental composition is assessed through FE-SEM and EDS analysis to support XRD and FTIR data. Further, the collaborative effect of an assimilated GO/Ce-based composite membrane is investigated systematically considering proton conductivity, water uptake and swelling ratio. The neutralization of the sulfonic acid groups presents in the perfluorosulfonated acid polymer shifted towards the lower side due to the indirect interaction provided by cerium ions; thus, the synthesized composite membranes show the optimum values of proton conductivity as compared to Nafion ^212^. Further, the antioxidative stability was tested by Fenton’s test for 24 h, showing better stability than that of pristine Nafion. The residual weight after the oxidative stability test demonstrated the excellent stability of the composite membrane towards the attack of radicals, proving the radical scavenging behavior of the hybrid material, which could be considered as a promising candidate for proton exchange membranes in long-term fuel cell applications.

## Figures and Tables

**Table 1:** Water uptake (WU), swelling ratio (SR), ion exchange capacity (IEC) and proton conductivity of different types of Nafion-GO/Ce membranes.

Membrane	WU (%)	SW (%)	IEC (meq g^−1^)	Conductivity (S·cm^−1^)
Nafion	19	09	0.90	0.068
Nafion-GO/Ce-1	21	11	0.82	0.059
Nafion-GO/Ce-2	23	13	0.78	0.061
Nafion-GO/Ce-3	20	08	0.70	0.043
Nafion-GO/Ce-4	16	06	0.62	0.039
